# Supplementary material for: Prognostic Abilities of Pre- and Post-Treatment Inflammatory Markers in Oral Squamous Cell Carcinoma: Stepwise Modelling
Source: Medicina (Kaunas). 2022 Oct 10;58(10):1426. doi: 10.3390/medicina58101426 (PMC9606996; doi:10.3390/medicina58101426)

Supplementary file S1: Kaplan–Meier graphs for OS and DFS of the markers that were identified as having significant prognostic abilities

Figure S1. Kaplan-Meier estimates for overall survival (OS), stratified by levels of lymphocyte-to-monocyte ratio (LMR)

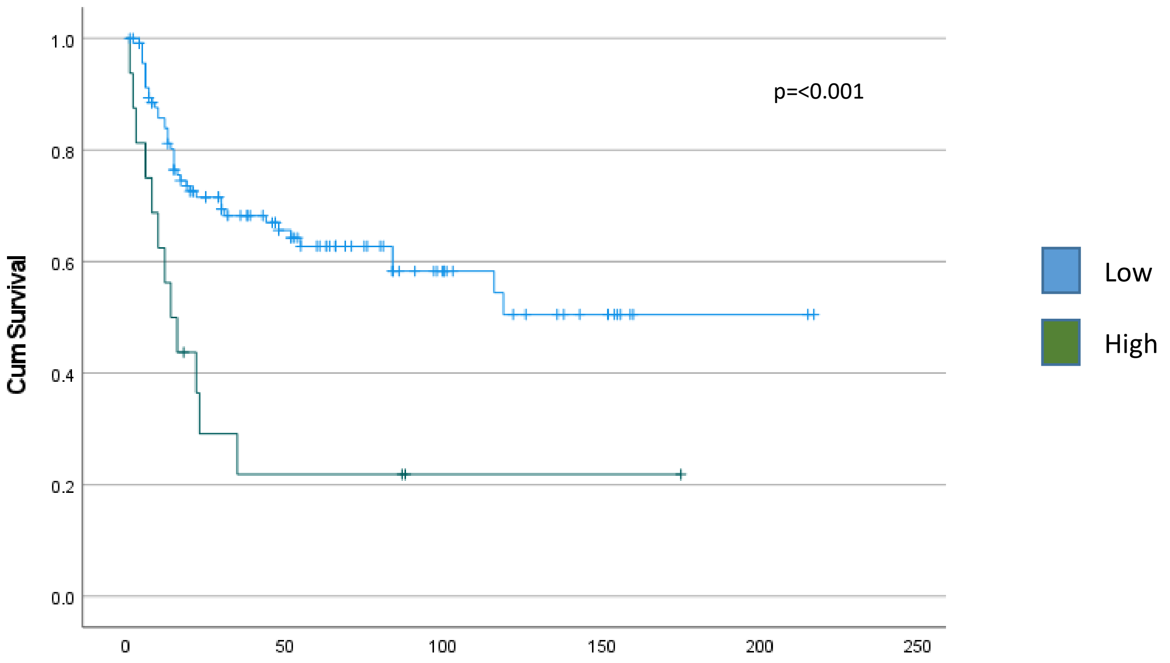

Figure S2. Kaplan-Meier estimates for overall survival (OS), stratified by levels of platelet count (PLC)

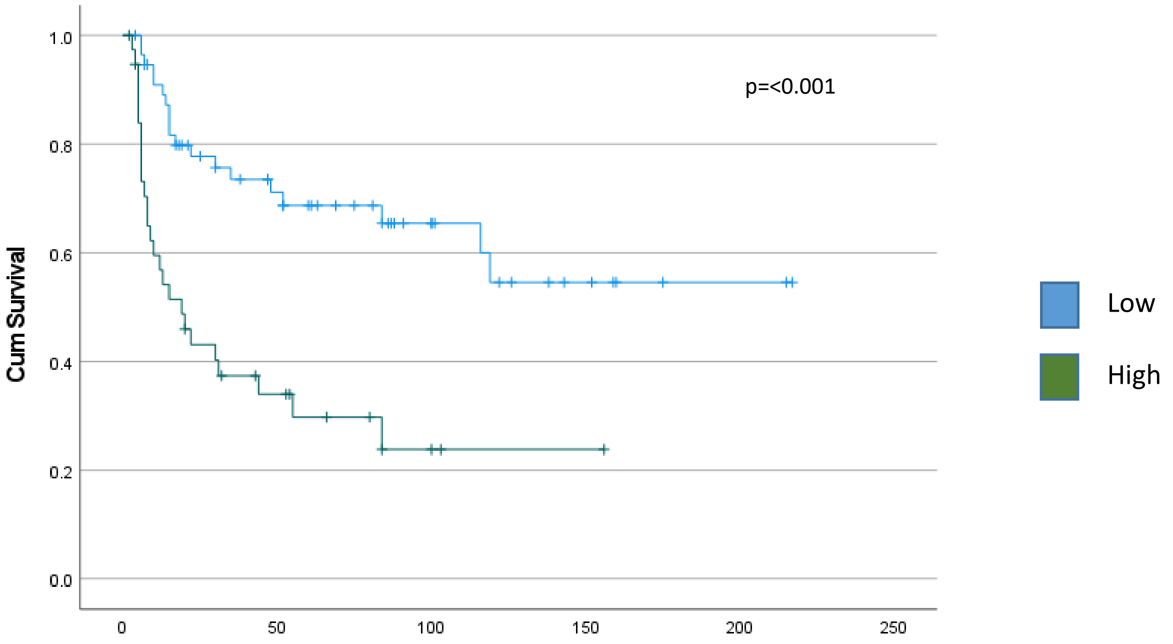

Figure S3. Kaplan-Meier estimates for overall survival (OS), stratified by levels of platelet-to-lymphocyte ratio (PLR)

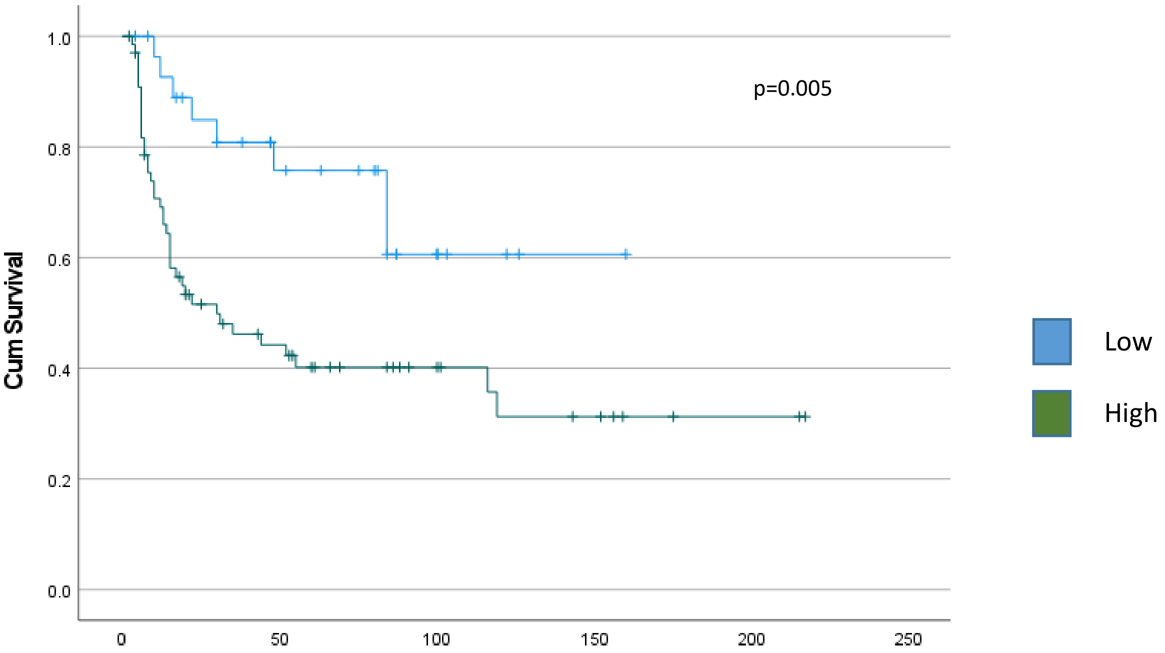

Figure S4. Kaplan-Meier estimates of disease-free survival (DFS), stratified by levels of systemic immune-inflammation index (SII)

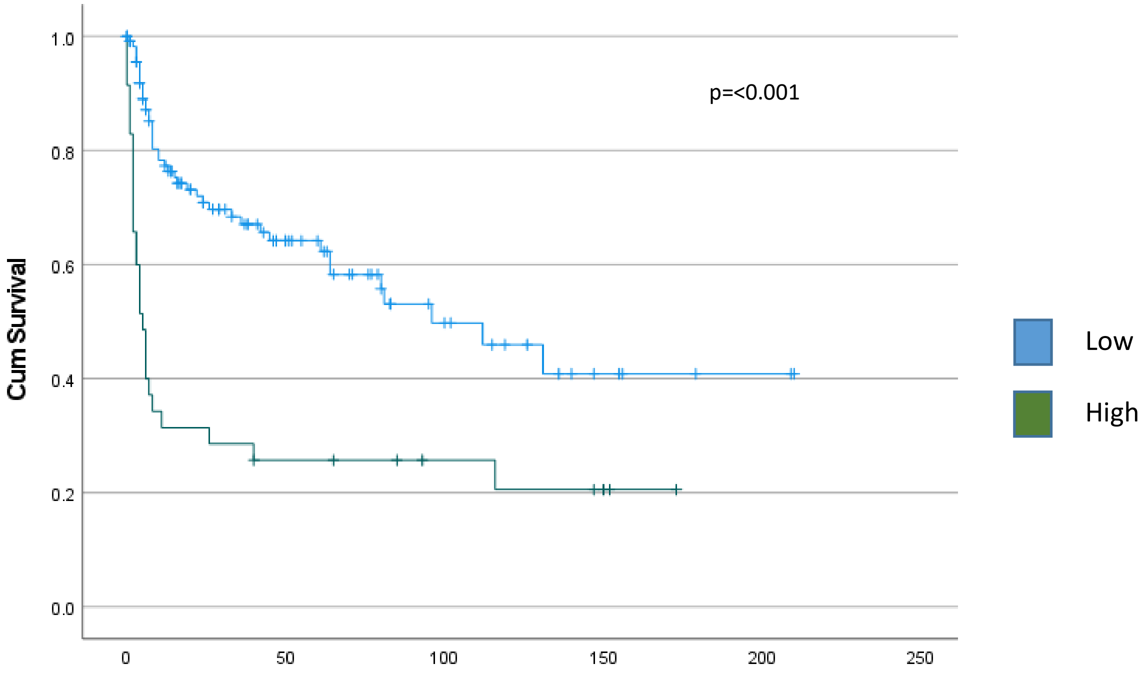

Figure S5. Kaplan-Meier estimates of disease-free survival (DFS), stratified by levels of platelet count (PLC)

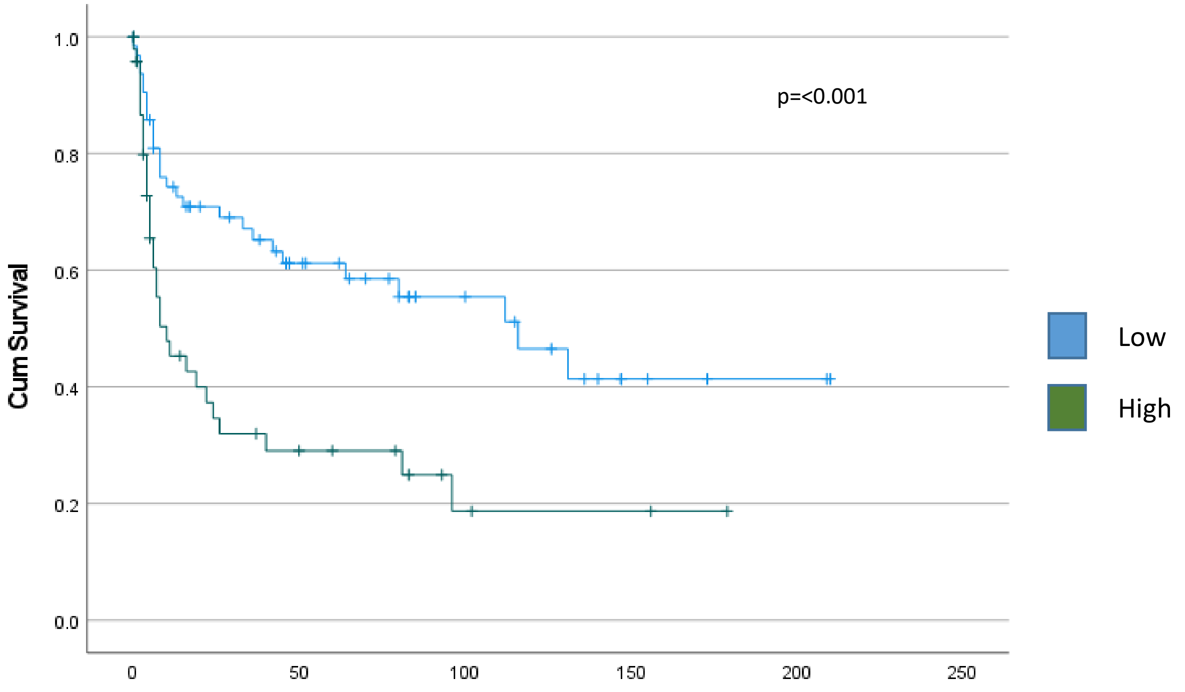

Figure S6. Kaplan-Meier estimates of disease-free survival (DFS), stratified by levels of platelet-to-lymphocyte ratio (PLR)

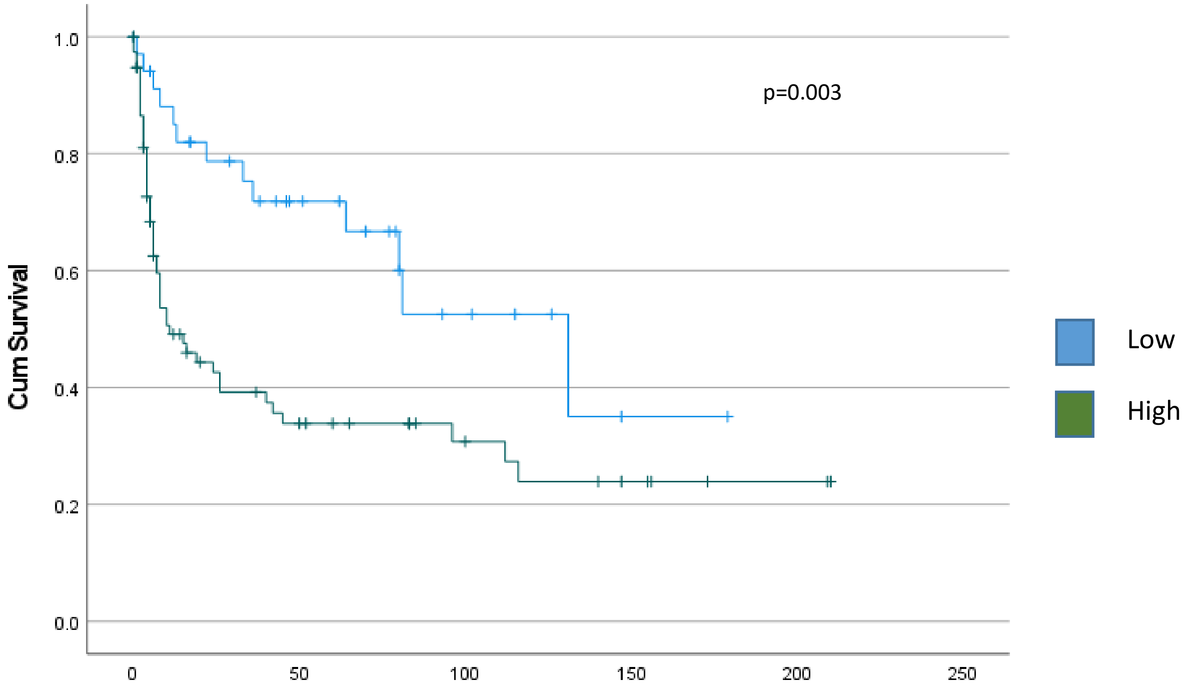

Supplement: Supplementary file 1 [file medicina-58-01426-s001.zip › medicina-1908325-supplementary.pdf]
